# Calcium (II) - and dipicolinic acid mediated-biostimulation of oil-bioremediation under multiple stresses by heat, oil and heavy metals

**DOI:** 10.1038/s41598-017-10121-7

**Published:** 2017-08-25

**Authors:** Samir S. Radwan, Dina M. Al-Mailem, Mayada K. Kansour

**Affiliations:** Microbiology Program, Department of Biological Sciences, Faculty of Science, Kuwait University, P O Box 5969, Safat, 13060 Kuwait

## Abstract

The oil-producing Arabian Gulf states have hot summer seasons of about 7-month in length. Therefore, environmental oil spills should be bioremediated by the activity of indigenous, hydrocarbonoclastic (hydrocarbon-degrading) microorganisms with optimum growth at about 50 °C. Soils in such arid countries harbor thermophilic bacteria, whose oil-consumption potential is enhanced by calcium (II) - and dipicolinic acid (DPA)-supplement. Those organisms are, however, subjected to additional stresses including toxic effects of heavy metals that may be associated with the spilled oil. Our study highlighted the resistance of indigenous, thermophilic isolates to the heavy metals, mercury (II), cadmium (II), arsenic (II) and lead (II) at 50 °C. We also detected the uptake of heavy metals by 15 isolates at 50 °C, and identified the *merA* genes coding for Hg^2+^-resistance in 4 of the studied Hg^2+^-resistant isolates. Hg^2+^ was the most toxic metal and the metal toxicity was commonly higher in the presence of oil. The addition of Ca^2+^ and DPA enhanced the Hg^2+^-resistance among most of the isolates at 50 °C. Crude oil consumption at 50 °C by 4 selected isolates was inhibited by the tested heavy metals. However, Ca^2+^ and DPA limited this inhibition and enhanced oil-consumption, which exceeded by far the values in the control cultures.

## Introduction

Globally, the Arabian Gulf states are major oil producers. Consequently, oil spills during oil production, transportation and use as an energy source are more frequent in that region than elsewhere. This region is an arid zone with a harsh climate. It is characterized by hot summer seasons, usually extending from mid-April to mid-October. This is also the case in several other oil-producing countries over the globe. Summer temperatures of about 50 °C and higher are usual in the Gulf region, leading to minimized activities of indigenous, mesophilic microorganisms that are responsible for oil bioremediation world-wide^1–4^. For bioremediation to occur under these hot conditions, thermophilic microorganisms (growing best at about 50 °C) with hydrocarbon degradation potential should be involved^5^.

Although there is currently a wealth of information on thermophilic microorganisms and their practical applications^6–9^, thermophiles with hydrocarbonoclastic potential have been only poorly investigated. The first report on a thermophilic bacterium capable of oil degradation appeared in the 1960^10^. Subsequently, it took a number of decades for more reports on this subject to appear^11–13^. Thermophilic bacteria with hydrocarbonoclastic potential reportedly belonged to the genera *Bacillus / Geobacillus*, *Thermomicrobium* and *Thermooleophilum*. Our group contributed to such studies at that time by a publication on a local *Bacillus stearothermophilus* strain capable of crude oil utilization at about 50 °C.

More recently, our group has published a comprehensive survey of 19 thermophilic hydrocarbonoclastic bacterial taxa indigenous to a desert soil in the Arabian Gulf region (Kuwait)^14^. The predominant species, which grew and consumed hydrocarbons at 50 °C better than at 30 °C, belonged to the genera; *Amycolatopsis, Chelativorans, Isoptericola, Nocardia, Aeribacillus, Aneurinibacillus, Brevibacillus, Geobacillus, Kocuria, Marinobacter* and *Paenibacillus*. Information about the temperature relations, growth requirements and genetic characteristics of those thermophiles is available in our earlier publication^14^. The diversity of those thermophilic bacteria may reflect a potential for spilled-oil removal at 50 °C. A new finding of that survey was that growth and hydrocarbonoclastic activities of all the tested organisms were enhanced in response to treating the cultures with Ca^2+^ and dipicolinic acid (DPA). Both substances are long known to accumulate in cells of *Bacillus* spp during endospore formation, and are believed to be involved in the heat-resistance mechanisms of the spores, probably by stabilizing DNA at elevated temperatures^15, 16^. These facts indicate that Ca^2+^- and DPA-amendment may enhance microbial tolerance to heat, and may thus stimulate their potential for spilled-oil bioremediation in the thermophilic range. As mentioned, we have already provided experimental evidence for the validity of this assumption^14^. However, the thermophilic, hydrocarbonoclastic isolates *in situ* have to operate under multiple stresses. In addition to standing stresses due to toxic, aromatic oil constituents^17–19^ and heat, the microorganisms should withstand the toxicity exerted by heavy metals associated with crude oil^20–22^. Therefore, we now have elaborated on our earlier study by focusing on the effects of crude oil and heavy metals on the microbial isolates. Furthermore, we have investigated the probable roles of Ca^2+^-and DPA-amendment in resisting the toxic effects of heavy metals that lead to reduced growth and hydrocarbon consumption activity of those thermophiles at 50 °C. The results are expected to be useful in designing biotechnologies for bioremediating environmental oil spills in hot countries.

## Results and Discussion

Nineteen thermophilic bacterial species with hydrocarbonoclastic potential were used; they had been isolated as pure cultures, and characterized by sequencing their 16 S rDNA regions as described earlier^14^. Four heavy metals, Hg^2+^, Cd^2+^, As^2+^ and Pb^2+^, were chosen for this study, based on their frequent use in earlier similar studies on mesophilic microorganisms^23^.

### Minimum inhibitory concentrations of heavy metals at 50 °C

Heavy-metal resistance/tolerance is measured either as “maximum tolerated concentrations” (MTC)^24–26^ or as “minimum inhibitory concentrations” (MIC)^23, 27, 28^. In this study, we determined the MIC values of four heavy metals for the 19 studied strains at 50 °C in the absence and presence of crude oil vapor. The results are presented in Table 1. As expected, the various bacterial species exhibited different MIC values depending on their identities and on the heavy metal used. In the absence of oil vapor, the MIC values were lowest with Hg^2+^ (<5–90 ppm) and highest with As^2+^ (70 − > 14,800 ppm), reflecting highest and lowest toxicities, respectively. Cd^2+^ with MIC values between 5 and 700 ppm was next to Hg^2+^ in toxicity to the tested microorganisms. *Kocuria rosea* (MIC of 5,600 ppm Cd^2+^) was exceptionally tolerant to this metal. With MIC-values between 50 and 4000 ppm, Pb^2+^ was relatively less toxic to most of the isolates than As^2+^. In many cases, the MIC values of the heavy metals were much lower in the presence of crude oil than in its absence, reflecting higher microbial sensitivity. Those differences were more pronounced with Hg^2+^, Cd^2+^ and Pb^2+^ than with As^2+^. However, exceptions were also recorded. For example, the MIC-values with *Bacillus niabensis* treated with Cd^2+^ was higher (>600 ppm) in the presence of oil vapor than in its absence (only 5 ppm). With As^2+^, the MIC values measured for *Amycolatopsis thermoflava, Chelativorans multitrophicus, Isoptericola variabilis and Nocardia farcinica* in the absence and presence of oil vapor were quite similar.

**Table 1:** Minimum inhibitory concentrations (MIC) [ppm] of heavy metals for 19 hydrocarbonoclastic bacteria at 50 °C on nutrient agar with and without oil vapor.

Bacteria	Minimal inhibitory concentrations of heavy metals, MIC (ppm)									
	On nutrient agar					On nutrient agar + oil				
	Hg	Hg + Ca^2+^	Cd	As	Pb	Hg	Hg + Ca^2+^	Cd	As	Pb
*Amycolatopsis thermoflava*	90	90	300	>14800	4000	20	20	50	>14800	500
*Bacillus carboniphilus*	30	30	50	>14800	140	<5	< 5	50	70	<10
*Bacillus firmus*	<5	10	700	>14800	4000	<5	<5	5	2000	<10
*Bacillus foraminis*	50	50	700	>14800	500	10	<5	5	2000	<10
*Bacillus licheniformis*	<5	90	5	2000	500	<5	<5	<5	70	<10
*Bacillus niabensis*	90	90	5	10000	500	10	<5	>600	70	<10
*Bacillus thermoamylovorans*	30	90	300	10000	500	<5	50	<5	70	<10
*Chelativorans multitrophicus*	50	170	700	>14800	1200	<5	20	>600	>14800	150
*Isoptericola variabilis*	30	50	50	>14800	1200	<5	20	50	>14800	150
*Nocardia farcinica*	10	20	50	>14800	1200	10	20	50	>14800	>1050
*Aeribacillus pallidus*	<5	50	50	>14800	1200	<5	<5	5	10000	150
*Aneurinibacillus danicus*	30	>210	50	>14800	500	10	10	5	2000	150
*Brevibacillus borstelensis*	<5	170	700	70	1200	<5	<5	50	70	150
*Brevibacillus thermoruber*	<5	10	700	70	50	<5	<5	50	70	<10
*Geobacillus kaustophilus*	<5	20	50	500	140	<5	30	50	70	<10
*Geobacillus subterraneus*	<5	10	700	5000	1200	<5	50	50	2000	>1050
*Kocuria rosea*	<5	10	>5600	10000	1200	<5	<5	50	5000	<10
*Marinobacter lutaoensis*	<5	10	50	70	50	<5	<5	50	70	<10
*Paenibacillus lautus*	<5	10	700	70	500	<5	<5	5	70	<10

Values are means of triplicates.

There are no relevant reports in the available literature on thermophiles (nor on mesophiles) with which to compare our findings. An explanation for the lower MIC heavy metal values in the presence rather than the absence of oil may be that oil exerts additional stress on the strains. Oil is known to contain polyaromatic hydrocarbons with toxic effects. Organisms whose MIC values were not affected by oil were probably active in biodegradation of the polyaromatic hydrocarbons. The results in Table 1 indicate that the heavy metals may commonly be arranged according to their toxicities to the studied isolates in the following order: Hg^2+^ > Cd^2+^ > Pb^2+^ > As^2+^. This toxicity order is more or less similar to that described by earlier workers on mesophiles^23^. A new finding in our study is that the amendment with Ca^2+^ as CaSO_4_, enhanced the resistance of many isolates to Hg^2+^ significantly (*p* < 0.05). This enhancement was particularly pronounced with *Bacillus licheniformis, B. thermoamylovorans, Chelativorans multitrophicus, Isoptericola variabilis, Aeribacillus pallidus, Aneurinibacillus danicus, Brevibacillus borstelensis, Geobacillus kaustophilus* and *G. subterraneus*. The two *Geobacillus* species in cultures that had been supplemented with with CaSO_4_ surprisingly tolerated Hg^2+^ in the presence of crude oil more effectively than in its absence. Again, there are no reports in the available literature on the resistance of microorganisms to heavy metals as affected by crude oil for comparison. This study thus provides some novel and important information in this area of research.

### Uptake of heavy metals at 50 °C

**Figure 1:**
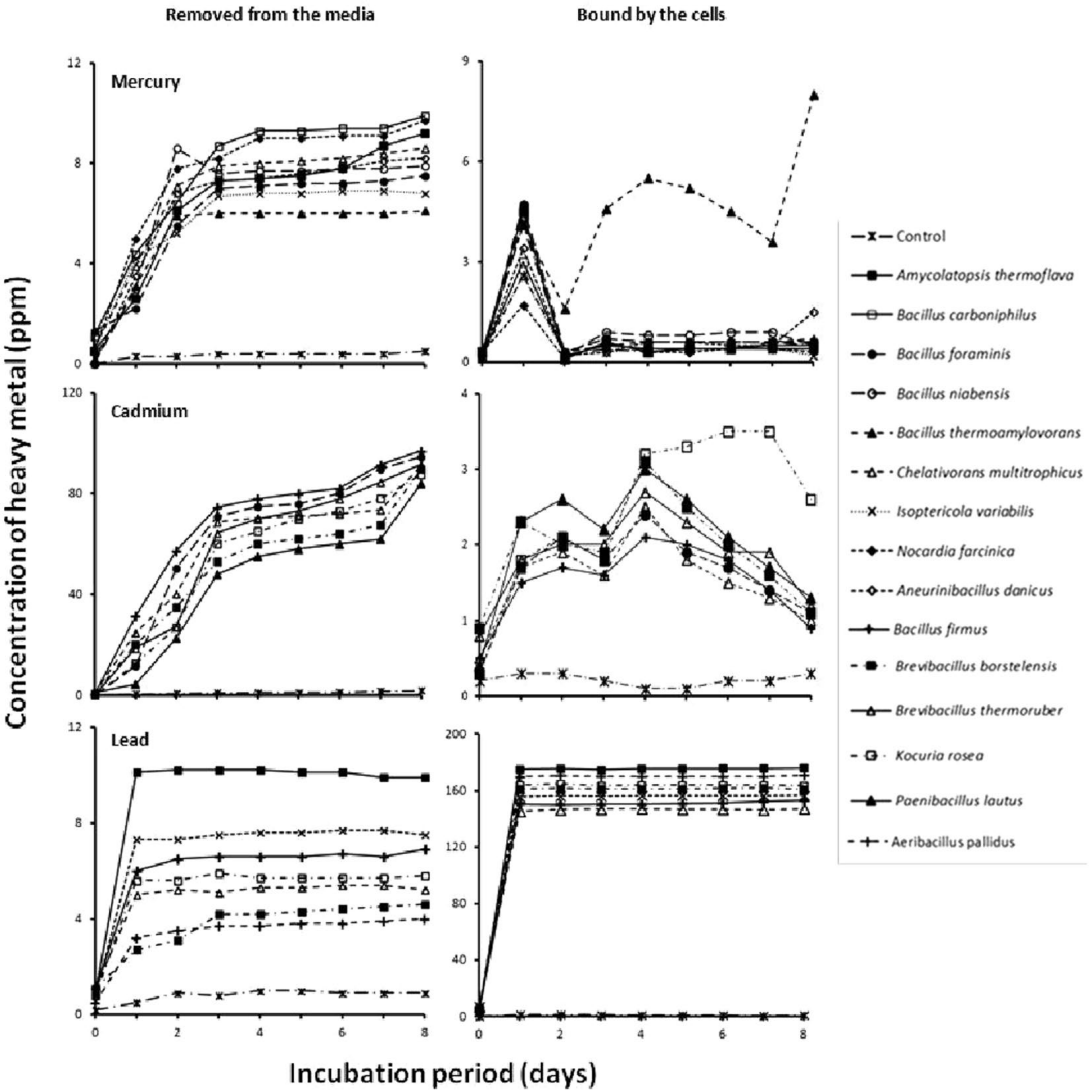
Uptake of heavy metal by hydrocarbonoclastic bacteria at 50 °C. Most of the amounts of the heavy metals added decreased in the media and accumulated in the cells by all the studied organisms during incubation. Each value was the mean of three parallel readings, and the deviation values were < 5.8% of the means.

To reduce the extensive set-up associated with this experiment, we selected the fifteen isolates that revealed (in preliminary experiments) high heavy metal resistance. The results in Fig. 1 show the uptake of Hg^2+^ as supplied by HgCl_2_, Cd^2+^ as CdSO_4_, and Pb^2+^ as Pb(NO_3_)_2_ by the studied hydrocarbonoclastic isolates at 50 °C. The four cations were taken up successfully by all the strains. Most of the Pb^2+^ was taken up from the medium relatively quickly, i.e. within the first day, and was maintained intracellularly through the 8 days of incubation. The uptake of Hg^2+^ and Cd^2+^, on the other hand, continued through the 8 day incubation period, although most of the cations were taken up during the first 3 days (left graphs). In most cases, the cells lost proportions of the bound Hg^2+^ and Cd^2+^ (but not Pb^2+^), at late phases of incubation (right graphs), yet not into the surrounding medium. It is well known that microorganisms have the potential for reduction of some heavy metals into volatile forms (e.g. Hg^2+^ into Hg)^23, 29–32^. Probably, *Bacillus thermoamylovorans* cells in the closed batch cultures (top-graph at the right, Fig. 1) again took up much of the volatilized Hg leading to increased amounts of cell-bounded Hg^2+^. In field treatment, the summer heat (about 50 °C or more) would enhance the volatilization process in the open atmosphere. This transformation (Hg^2+^ reduction to volatile Hg) is mediated by enzymes encoded by *merA* genes^33^. Therefore, our Hg^2+^-resistant species (based on preliminary experiments, see also Table 1) were analyzed for those genes.

**Figure 2:**
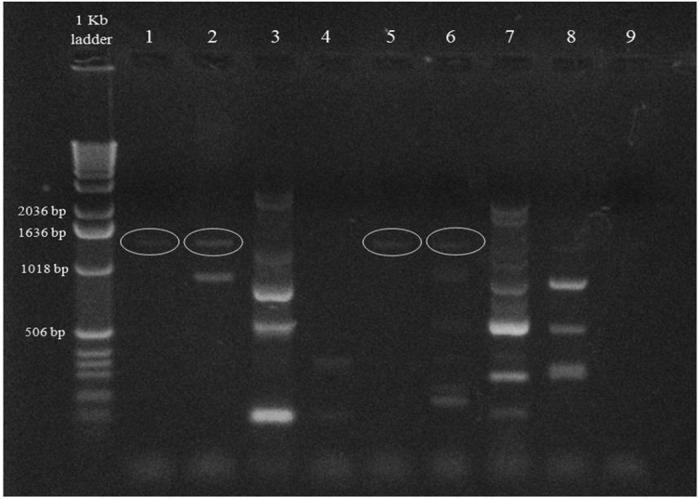
Electrophoresis profiles on agarose gel showing the PCR products (*merA* genes) in total genomic DNA from nine mercury resistant thermophilic bacteria. The primer pair used for amplification was Mer A3-f/ Mer A3-r. Bands of 1246 bp (in circles) were extracted, amplified, sequenced and the sequences compared with those in the GenBank database. The results of this sequencing are available in Table 2. 1, *Amycolatopsis thermoflava;* 2*, Bacillus carboniphilus;* 3*, Bacillus foraminis;* 4*, Bacillus niabensis;* 5*, Bacillus thermoamylovorans;* 6, *Chelativorans multitrophicus;* 7*, Isoptericola variabilis;* 8*, Nocardia farcinica;* 9*, Aneurinibacillus danicus*.

The results in Fig. 2 and Table 2 offer experimental evidence for the occurrence of *merA* genes in the genomes of four of the studied Hg^2+^-resistant isolates: *Amycolatopsis thermoflava, Bacillus carboniphilus, B. thermoamylovorans* and *Chelativorans multitrophicus*. It was not possible to identify those genes in another five Hg^2+^-resistant species, namely *B. foraminis, B. niabensis, Isoptericola variabilis, Nocardia farcinica* and *Aneurinibacillus danicus*. This failure was probably due to the well known diversity of the *merA* genes^34^, and to the unsuitability of the used primer sets for their common amplification. New primer sets for this purpose may will be constructed in the future.

**Table 2:** Information related to sequencing of *MerA* genes in the genomes of 9 Hg^2+^ resistant bacterial species (see Fig. 2).

Bacterial isolate	Primers used	Total bases	Nearest genebank match	Similarity %	Bases compared	Genebank accession no.
*Amycolatopsis thermoflava*	MerA3-F/ MerA3-R	897	Uncultured bacterium clone SS2–2 mercuric reductase *merA* gene	99	903/906	
*Bacillus carboniphilus*	MerA3-F/ MerA3-R	747	Uncultured bacterium clone SS2–13 mercuric reductase *merA* gene	99	749/750	
*Bacillus foraminis*	*MerA* gene not detected					
*Bacillus niabensis*	*MerA* gene not detected					
*Bacillus thermoamylovorans*	MerA3-F/ MerA3-R	790	Uncultured bacterium clone SS3–10 mercuric reductase *merA* gene	100	790/790	
*Chelativorans multitrophicus*	MerA3-F/ MerA3-R	504	Uncultured bacterium clone SS3–10 mercuric reductase *merA* gene	100	504/504	
*Isoptericola variabilis*	*MerA* gene not detected					
*Nocardia farcinica*	*MerA* gene not detected					
*Aneurinibacillus danicus*	*MerA* gene not detected					

### Consumption of crude oil at 50 °C

**Figure 3:**
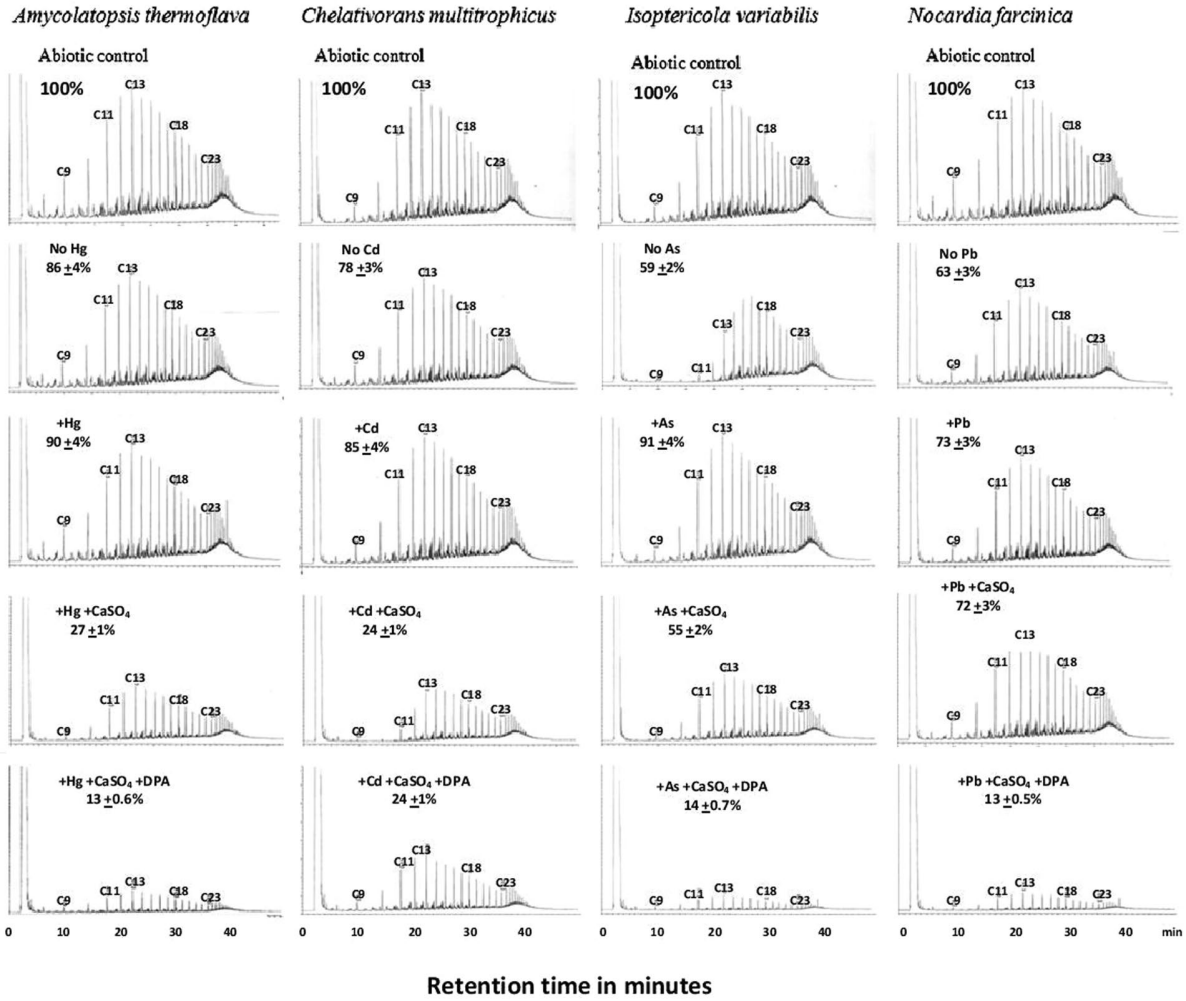
GLC-Profiles of residual crude oil showing the effects of Ca^2+^ and dipicolinic acid (DPA) on oil-consumption by bacteria at 50 °C in the presence of heavy metals. Percent values on the individual profiles are those of residual oil. Each value is the mean of 3 parallel analyses ± standard deviation. All changes in the oil concentrations compared to the 100% controls were significant with *p* < 0.05.

Due to the extensive work needed for this analysis, we selected, as representative species, the four isolates with the best growth potential (in preliminary experiments) in a mineral medium with oil as a sole source of carbon and energy^35^. The results in Fig. 3 show, after 2 weeks at 50 °C in the absence of heavy metals, that the four tested isolates consumed significant ( < 0.05) proportions of the crude oil available in the medium. *Isoptericola variabilis* and *Nocardia farcinica* with consumption values of 41 and 37%, respectively, were most effective, whereas *Chelativorans multitrophicus* and *Amycolatopsis thermoflava* with consumption values of 22 and 14%, respectively, were least effective. The oil-consumption values by the tested organisms decreased in the presence of the four tested heavy metals. In all cases, the consumption values were significant, with *p* < 0.05. The GLC profiles show that individual oil constituents (individual peaks) were consumed rather evenly by all the tested microorganisms.

An interesting and new finding is that the amendment with CaSO_4_ alone or in combination with DPA significantly (*p* < 0.05) stimulated oil consumption at 50 °C, even in the presence of inhibitory concentrations of heavy metals (see Methods, Uptake of heavy metals). In response to such amendments, the consumption values reached 87% of the amount available at time zero. From a biotechnological viewpoint, this result is quite impressive. However, the biochemical basis for the mechanisms of such biostimulation is still far from clear. In the available literature, there are no related studies with which to compare our data. However, endospores of *Bacillus* spp, are heat- and stress-resistant, like the studied thermophiles, and are known to contain DPA complexed with Ca^2+^ at about 15% of their dry weights^15^. Calcium-dipicolinate has been reported to stabilize nucleic acids and proteins (enzymes) at high temperature^16^. Should this be commonly the case, this interpretation could also be adopted to the effects of Ca^2+^ and DPA on the active cells of thermophilic, hydrocarbonoclastic bacteria, most of which are actually endospore-producers. As endospores, they would remain of course dormant without hydrocarbonoclastic or any other activities.

In conclusion, although exposed to multiple stresses (toxic oil-constituents, namely the polyaromatics, toxic heavy metals, heat), thermophilic, hydrocarbonoclastic bacteria indigenous to hot regions are still capable of consuming spilled oil. Their oil bioremediation potential can be effectively biostimulated by amendment with Ca^2+^ and DPA. These novel findings should be considered in designing biotechnologies for controlling oil-spills in hot regions. Based on the results of this contribution, the thermophiles, *Amycolatopsis thermoflava, Chelativorans multitrophicus, Isoptericola variabilis* and *Nocardia farcinica* may be suggested for bioremediation of oily soils under multiple stresses. These organisms may be biostimulated by amendment with Ca^2+^ and DPA.

## Methods

### Thermophilic bacteria

The nineteen hydrocarbonoclastic, thermophilic bacterial species used in this study are listed in Table 2. They had been isolated from oil-contaminated soil samples from Kuwaiti oil fields on a selective medium at 50 °C. This is a mineral medium with oil vapor as the sole source of carbon and energy^35^. It consisted of (g l^−1^): 5.0 NaNO_3_, 0.6 KH_2_PO_4_, 0.9 Na_2_HPO_4_, 0.2 K_2_SO_4_, 0.4 MgSO_4_ 7H_2_O, 0.7 CaCl_2_.2H_2_O, 2.5 ml of trace element mixture (g l^−1^): 2.3 ZnSO_4_, 1.8 MnSO_4_, 0.6 H_3_BO_3_, 1.0 CuSO_4_, 0.4 Na_2_ MoO_4_, 0.4 CoCl_2_, 0.7 KI, 1.0 EDTA, 0.4 FeSO_4_, 0.004 NiCl_2_, and 20.0 g agar. Oil vapor was made available by fixing in the dish lids filter papers impregnated with 2 ml crude oil and sealing the dishes. The isolated strains were purified and characterized by comparing the sequences of their 16 S rRNA-coding genes with those of strains in the GenBank database. Detailed information of these organisms is available in our earlier publication^14^.

### Uptake of heavy metals

A common inoculum was prepared for each tested strain by homogenizing a loopful of 48-h biomass in 10 ml sterile water. One ml of this suspension was inoculated into 200 ml nutrient broth (g l^−1^ :5.0 peptone, 2.0 yeast extract) provided with either 15 ppm Hg^2+^ as HgCl_2_, 112 ppm Cd^2+^ as CdCl_2_ or 207 ppm Pb^2+^ as Pb(NO_3_)_2_. Three parallel replicates were prepared for each analysis. Cultures were incubated at 50 °C on an electrical shaker, 100 rpm, for 8 days. From each culture, 10 ml were taken at time zero and then daily. The aliquots were centrifuged at 6000 × *g* for 10 min at 4 °C, and the pellets were washed 3 times with nutrient broth. The washed pellets, the combined supernatants and 1 ml of the control (cell-free medium containing the heavy metal) were quantitatively analyzed for the heavy metals. In experiments with Hg^2+^, biomass and supernatant samples were first digested with a mixture of concentrated HNO_3_ and HCl, 2:1, v/v, on a hot plate. The digested solutions were filtered, diluted with 1% HNO_3_ and the total mercury concentrations were measured by an inductively coupled plasma-atomic emission spectrophotometer (ICP-AES; Thermo Elemental, Franklilakes, NJ, USA, or JY 2000 Ultrace, Jobin–Yvon Horiba) using a certified reference soil standard containing mercury solution. For Cd^2+^ and Pb^2+^ an inductively coupled plasma mass spectrophotometer (ICP-MS, varian-820-MS, NJ, USA) was used to measure the concentrations directly. The percent values of heavy metals remaining in the medium were calculated.

### The *merA* gene analysis

Total genomic DNA was extracted from 24-h bacterial biomass using a PrepMan Ultra Kit (Applied Biosystems, Foster City, CA, USA). The merA region was amplified by PCR using 9 different primer pairs (sequences are listed in Table 3). The reaction mixture contained puReTaq Ready-To-Go PCR Beads (Amersham Biosciences, UK), 1 µl (25 ng) of DNA template and 1 µl each of the primer combinations. The reaction volume was completed to 25 µl with molecular water. Amplification was carried out in a Veriti Thermal Cycler (Applied Biosystems, USA) and the PCR conditions were 94 °C for 2 min, followed by 35 cycles of 94 °C for 1 min, 50–64 °C for 30 sec, 72 °C for 1–3 min and one cycle of final extension at 72 °C for 10 min (Table 3). The amplicons were resolved by electrophoresis on agarose gel plates, and bands were purified using a QIA gel extraction kit (Qiagen, USA). Sequencing of amplicons was performed by a BigDye version Terminator Kit (Applied Biosystems, USA); 20 ng of the DNA template was added to 2 μl of a BigDye v 3.1 terminator and 2 μl of BigDye Terminator v 1.1, v 3.1 5x sequencing buffer; l μl of either the forward or reverse primer was added, and the final volume was brought up to 10 μl with molecular water. Labeling was completed in a Veriti Thermal Cycler (Applied Biosystems, USA) using one cycle of 96 °C for l min, then 25 cycles of l min at 96 °C, 5 s at 50 °C and 4 min at 60 °C. The pure template DNA samples were processed in a 3130xl Genetic Analyzer (Applied Biosystems, USA). Sequencing analysis version 5.2 software (Applied Biosystems, USA) was used to analyze the results. Sequences were subjected to basic local alignment search tool analysis with the National Center for Biotechnology Information (NCBI; Bethesda, MD, USA) GenBank database^36^.

**Table 3:** Primer sets used for *merA* gene amplification^33^.

Primer	Sequence (5′−3′)	Length	Annealing temp. (°C)	Ext. time (min)
Mer A3-f	CGTSAACGTSGGSTGCGTGCCSTCCAAG	1246	64	3
Mer A3-r	CGAGCYTKARSSCYTCGGMCAKSGTCA			
Act-Fw	CSGAVTTGGTSTACGTCGC	397	62	1
Act-Rv(a)	ATGAGGTASGGG			
Al-Fw	TCCAAGGCGMTGATCCGCGC	~800	63	1.5
Al-Rv	TAGGCGGCCATGTAGACGAACTGGTC			
Als-n-F	TCCGCAAGTNGCVACBGTNGG	1329–1638	62	3
MerA5-R	CGCYGCRAGCTTYAAYCYYTCRRCCATYGT			
Umer A-F	CTGGTTGTGAAGAACAT	1556	50	3
Umer A-R	TCCTTCTGCCATTGTTA			
Fir-Fw	GTTTATGTWGCWGCYTATGAAGG	455	64	1
Fir-Rev 1892	CCTGCACARCAAGATAATTTBGA			
Fir-Fw	GTTTATGTWGCWGCYTATGAAGG	455	64	1
Fir-Rev 1832	CCTTCWGCCATYGTTARATAWGG			
Act-Fw	CSGAVTTGGTSTACGTCGC	397	62	1
Act-Rv(b)	GCCATGAGGTASGGG			
A7s-n-F	CGATCCGCAAGTGGCIACBGT	288	60	1
55-n-R	ACCATCGTCAGRTARGGRAAVA			

### Heavy metal toxicity

To determine the minimum inhibitory concentrations (MIC) of the heavy metals, 10 μl aliquots of a cell suspension (48-h biomass in 5 ml water) of the tested strain were spot inoculated on nutrient agar containing a concentration gradient range for each heavy metal based on results of preliminary experiments: 0 to 210 ppm Hg^2+^, 0 to 5600 ppm Cd^2+^, 0 to 14800 ppm As^2+^ and 0 to 4400 ppm Pb^2+^. To study the effect of crude oil on the MIC values, the experiment was repeated using nutrient agar plates exposed to oil vapor, as described above. The effect of CaSO4 (2.5 M) and DPA (8% w/v) on the MIC values using both media was also studied. Triplicates were done throughout and the inoculated cultures were incubated at 50 °C for 3 days. They were examined for growth and the lowest heavy metal concentration at which growth ceased (MIC) was recorded.

### Crude oil consumption

The oil sample used was Kuwaiti light crude, which according to the Kuwaiti oil company consisted of 60% aliphatics, 29% aromatics, 8% asphalins and 3% resins. To measure the oil-consumption by the tested bacteria, 1 ml-aliquots of cell suspensions (loopful of 48-h biomass in 10 ml water) were inoculated in 250 ml flasks containing 100 ml mineral medium^32^ provided with 0.3% (w/v) light crude as the sole source of carbon and energy. Control flasks were prepared similarly but were inoculated with previously autoclaved cell suspensions. The oxygen tension in the media (as measured by Orion Star Meter, Thermo Scientific, USA) was 6.7 + 0.1 mgl^−1^. The flasks were sealed (to avoid oil loss by volatilization) and shaken at 100 rpm and 50 °C. To study the effect of Ca^2+^ and DPA amendment on the oil consumption, additional sets of flasks were prepared and treated either with 2.5 M CaSO_4_ or 2.5 M CaSO_4_ + 8% w/v DPA; these concentrations were selected based on results of preliminary experiments. Three replicate flasks were prepared throughout. Whole flask contents were harvested after 2 weeks. Residual hydrocarbons were extracted with three successive 15-ml aliquots of pentane. The combined extract was completed to 50 ml using pentane and 1 ml was analyzed by gas liquid chromatography (GLC). The GLC analysis was done using a Chrompack CP-9000 instrument equipped with a FID (Chrompack Middelburg, The Netherlands), a WCOT-fused silica CP-SIL-5 CB capillary column (Chrompack), and a temperature program of 45–310 °C with temperature rising 10 °C min^−1^ using N_2_ as a carrier gas. The detector temperature was 300 °C and the injector temperature 270 °C. The percentage of hydrocarbons remaining in the media was calculated as the percentage of total hydrocarbon peak areas based on the areas of peaks of hydrocarbons recovered from the previously autoclaved controls. The mean values of the 3 replicates and the standard deviation values were calculated.

### Statistical analysis

As already mentioned, all the readings were means of 3 replicates. Mean values of the triplicates ± standard deviation values were calculated using Microsoft Excel 2007. Statistical Package of Social Science, version 12, was used to assess the degree of significance, where the *t*-test and analysis of variance were used to differentiate between the means of the tested parameters.
